# Dietary patterns associated with hypertension risk among adults in Thailand: 8-year findings from the Thai Cohort Study

**DOI:** 10.1017/S1368980018002203

**Published:** 2018-09-06

**Authors:** Zumin Shi, Keren Papier, Vasoontara Yiengprugsawan, Matthew Kelly, Sam-ang Seubsman, Adrian C Sleigh

**Affiliations:** 1 Department of Human Nutrition, Qatar University, PO Box 2713, Doha, Qatar; 2 Adelaide Medical School, University of Adelaide, Adelaide, Australia; 3 South Australia Health and Medical Research Institute, Adelaide, Australia; 4 National Centre for Epidemiology and Population Health and Department of Global Health, Research School of Population Health, ANU College of Health & Medicine, The Australian National University, Canberra, Australia; 5 Population Health Department, QIMR Berghofer Medical Research Institute, Brisbane, Australia; 6 Centre for Research on Ageing, Health, and Wellbeing, The Australian National University, Canberra, Australia; 7 Thai Health-Risk Transition Study, School of Human Ecology, Sukhothai Thammathirat Open University, Nonthaburi, Thailand

**Keywords:** Dietary patterns, Factor analysis, Hypertension, Blood pressure, Cohort study, Thailand, South-East Asia

## Abstract

**Objective:**

Dietary intake is a leading risk factor for hypertension. We aimed to assess longitudinal associations between overall dietary patterns and incident hypertension among adults in Thailand.

**Design:**

Prospective large Thai Cohort Study (TCS) conducted nationwide from 2005 to 2013. Dietary patterns were identified using factor analysis based on usual intake of fourteen food groups. Multivariable logistic regression assessed associations between dietary patterns and hypertension prevalence and incidence.

**Setting:**

Emerging hypertension and changing diets in Thailand.

**Subjects:**

TCS participants who were normotensive at baseline in 2005.

**Results:**

Among 36293 participants without hypertension at baseline, 1831 reported incident hypertension (5·1 % incidence) at follow-up. Two dietary patterns were identified: ‘Modern’ and ‘Prudent’. The Modern dietary pattern (high intakes of roasted/smoked foods, instant foods, canned foods, fermented fruits/vegetables, fermented foods, soft drinks, deep-fried foods) was associated with increased incident hypertension (comparing extreme quartiles, OR for incident hypertension=1·51; 95 % CI 1·31, 1·75 in 2013). The Prudent dietary pattern (high intakes of soyabean products, milk, fruits, vegetables) was not associated with incident hypertension in a fully adjusted model. The association between the Modern dietary pattern and hypertension was attenuated by BMI.

**Conclusions:**

Modern dietary pattern was positively associated with hypertension among Thai adults. BMI had a great impact on the relationship between the Modern dietary pattern and incidence of hypertension. Reduction of Modern diets would be expected to prevent and control hypertension. Such a strategy would be worth testing.

Hypertension is one of the most important contributors to premature death worldwide^(^ ^1^ ^)^. Globally, dietary intake has been implicated as a leading risk factor for hypertension. For example, numerous studies have confirmed that high intake of Na and low intake of K increase the risk of hypertension^(^ ^2^ ^)^. As well, low intakes of vegetable protein^(^ ^3^ ^)^, Mg^(^ ^4^ ^)^, P^(^ ^5^ ^)^ and PUFA^(^ ^6^ ^)^, and higher intakes of soft drinks^(^ ^7^ ^)^, cholesterol^(^ ^8^ ^)^, animal protein^(^ ^3^ ^)^ and red meat^(^ ^9^ ^)^, have also been shown to be related to hypertension. Accumulating evidence from randomized clinical trials has shown that vegetarian diets, and those low in saturated animal fat and high in dietary fibre such as the Dietary Approaches to Stop Hypertension (DASH) diet, can effectively lower blood pressure^(^ ^2^ ^,^ ^10^ ^)^.

Analysing the association between overall dietary pattern and health outcomes is becoming more common as it may account for cumulative and interactive effects and control for dietary confounding^(^ ^11^ ^)^. However, the association between overall dietary patterns and hypertension has not been thoroughly studied and the findings are inconclusive^(^ ^2^ ^,^ ^10^ ^,^ ^12^ ^–^ ^14^ ^)^. Diet is largely determined by regional food availability, culture and distribution. To design effective dietary approaches to prevent hypertension, it is important to understand the specific association between dietary patterns and hypertension in specific settings.

Information on the association between dietary patterns and hypertension is limited in South-East Asia. This includes Thailand, which urgently needs preventive information to respond to its emerging hypertension epidemic. The Fourth Thai National Health Examination Survey (2008–2009) found associations between dietary patterns and risk of hypertension, but these data were cross-sectional^(^ ^15^ ^)^. In the Thai Cohort Study (TCS), another nationwide study with substantial data on nutrition and hypertension, longitudinal food-based analyses to date have shown that a high intake of fast foods was associated with incident hypertension between 2005 and 2009^(^ ^16^ ^,^ ^17^ ^)^. To expand our TCS analysis on this topic and to contribute to the need for longitudinal research, we have now identified dietary patterns and their association with hypertension using the 8-year prospective TCS data. The analysis addresses the knowledge gap on dietary patterns and hypertension in Thailand.

## Methods

### Study participants

The TCS is an ongoing population-based study. Three survey waves have been conducted in 2005, 2009 and 2013. The study recruited distance-learning adult students enrolled at Sukhothai Thammathirat Open University. At baseline (2005), 87151 cohort members were enrolled and were found to reside nationwide and to be broadly representative of the Thai population both socio-economically and geographically. The details of TCS recruitment and methodology have been published elsewhere^(^ ^17^ ^)^. Self-administered questionnaires were used to collect detailed information on sociodemographic characteristics, childhood environment, transport and injury, illness and health-service use, lifestyle and health information at all waves. Informed written consent was obtained from all participants. The first follow-up in 2009 reached 60569 (70 %) of all baseline participants and the second follow-up in 2013 reached 42785 (71 % of 2009 participants).

### Eligibility

Participants were those who did not have missing dietary intake data in 2005, did not have hypertension in 2005 and who reported a hypertension status in 2013.

### Outcome variable: hypertension

At each wave, participants were asked whether they were told by a doctor that they had hypertension. Incident hypertension was defined as normotensive in 2005 baseline but hypertensive in 2013. Previously, a validation study established that the accuracy of self-reported hypertension was high, with a sensitivity and specificity of 82·4 and 70·7 %, respectively^(^ ^18^ ^)^.

### Exposure variables: dietary intake

At baseline survey (2005), intake of fruits (and vegetables) was determined by the question, ‘How many servings of fruits (vegetables) do you usually eat each day?’. In addition, habitual intake of twelve food groups (foods/desserts with coconut milk; deep-fried foods; fermented foods; roasted/smoked foods; uncooked meat or shrimp; fermented fruits/vegetables; instant foods; canned foods; soft drinks; milk; soyabean products; food supplements, vitamins and minerals) was ascertained by the questions, ‘How often, on average, do you eat each of the following foodstuffs? (i) Never or less than once a month, (ii) 1–3 times/month, (iii) 1–2 times/week, (iv) 3–6 times/week, (v) once a day or more’. The intake frequencies of the above twelve food groups were recoded as d/week: 0, 0·5, 1·5, 4·5 and 7, respectively.

### Covariates

Information on sociodemographic and lifestyle factors in each wave was collected using a structured questionnaire. In the analyses, the following constructed variables were used as indicators of socio-economic status: education (junior high school; high school; diploma/certificate; university degree) and personal monthly income (recoded into tertiles as low (up to 7000 Baht), medium (7000–20000 Baht) and high (>20000 Baht)). If a variable had missing values, a specific category was assigned instead of excluding the record.

Frequency of taking leisure-time physical activity (strenuous exercise, moderate exercise or mild exercise for more than 20 min, walking for at least 10 min) was recoded as times/week (for each type of activity).

Smoking status was categorized as never smoked, current smoker, ex-smoker, ever smoked but status unclear, and unknown.

Height and weight were reported at each wave. BMI was calculated as weight in kilograms divided by the square of height in metres.

### Statistical analysis

Dietary patterns at baseline based on the above fourteen food groups (twelve food categories and fruits and vegetables) were identified using principal component analysis. Factors were rotated with an orthogonal (varimax) rotation to improve interpretability and minimize the correlation between the factors. We used eigenvalues (>1·0), a scree plot and factor interpretability to determine the number of factors to retain. Labelling of the factors was primarily descriptive based on our interpretation of the pattern structures and on the food items with the highest factor loadings. Factor loadings were graphically presented. Factor loadings are equivalent to correlations between the food items and the factor. Higher loadings (absolute value) indicate that the food shares more variance with that factor. Factor scores were calculated as the sum of the product of the standardized intake of each individual food item and its factor loading.

Dietary pattern scores were recoded into quartiles. The *χ* ^2^ test was used to compare differences between groups for categorical variables and ANOVA for continuous variables. We used logistic regression to assess the associations between dietary patterns and hypertension and incidence in 2013. We estimated OR and 95 % CI. Three models were used: model 1 adjusted for age and sex; model 2 further adjusted for smoking, alcohol drinking, income, education and physical activity; and model 3 further adjusted for BMI.

As there was no significant interaction between dietary patterns and sex, we did not present sex-specific analyses. We recoded missing values as a specific group in the analyses. The association (*β* and 95 % CI) between dietary patterns and BMI was visually presented after running a linear regression adjusted for age and sex. A linear trend was tested by using the original dietary pattern scores as continuous variables in the regression model. All analyses were performed using the statistical software package Stata version 15.1. Statistical significance was considered when *P*<0·05 (two sided).

## Results

Of the 87151 initial TCS participants, 81723 participants (36882 men and 44838 women, three had missing values) had valid dietary intake data and hypertension data in 2005. Among these participants, 3698 had prevalent hypertension (4·5 %) and were excluded from further analyses. Of the remaining 78025, a total of 36293 eligible participants (without missing hypertension responses) were followed-up in 2013. The final study sample included 36293 participants of whom 1831 reported incident hypertension (5·1 % incidence). The mean age was 30·5 (sd 8·2) years at baseline.

### Dietary patterns

**Fig. 1:**
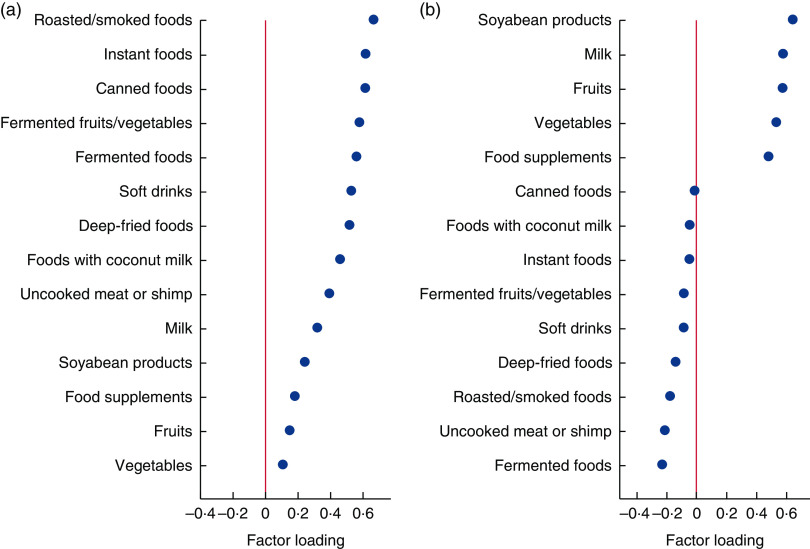
(colour online) Factor loadings of baseline dietary patterns identified in Thai Cohort Study participants (*n* 81725): (a) Modern dietary pattern; (b) Prudent dietary pattern

Factor analysis revealed two dietary patterns. The ‘Modern’ dietary pattern had high loadings (>0·30 or <−0·30) for roasted/smoked foods, instant foods, canned foods, fermented fruits/vegetables, fermented foods, soft drinks, deep-fried foods, foods/desserts with coconut milk and uncooked meat or shrimp (Fig. 1). The ‘Prudent’ dietary pattern had high loadings for soyabean products, milk, fruits, vegetables and food supplements, with inverse loadings for fermented foods and uncooked meat or shrimp. The two dietary patterns explained 21·2 and 12·5 % of the variance in food intake, respectively.

The Modern dietary pattern was inversely associated, and the Prudent dietary pattern was positively associated, with high education. Men were more like to have high intake of the Modern dietary pattern while women were more likely to have high intake of the Prudent dietary pattern (Table 1). After adjusting for age and sex, the Modern dietary pattern was positively associated with BMI and the Prudent dietary pattern was inversely associated (*P* trend<0·001; see online supplementary material, Supplemental Fig. 1). Comparing extreme quartiles of dietary pattern scores, the regression coefficients for BMI in 2005 were 0·39 (95 % CI 0·33, 0·46) for the Modern dietary pattern and −0·48 (95 % CI −0·54, −0·41) for the Prudent dietary pattern.

**Table 1:** Baseline characteristics by quartiles of dietary patterns for eligible participants in the Thai Cohort Study (*n* 81725)

	Dietary pattern by quartile of intake									
	Modern					Prudent				
Sociodemographic characteristic	Q1	Q2	Q3	Q4	*P* value*	Q1	Q2	Q3	Q4	*P* value*
(*n* 20432)	(*n* 20431)	(*n* 20431)	(*n* 20431)	(*n* 20432)	(*n* 20431)	(*n* 20431)	(*n* 20431)
Age (years)										
Mean	33·5	30·7	29·3	28·1	<0·001	31·3	30·2	30·0	30·1	<0·001
sd	9·1	8·0	7·5	7·0		8·1	8·0	8·2	8·4	
Sex (%)					<0·001					<0·001
Male	38·3	44·4	47·6	50·1		59·4	47·0	39·7	34·4	
Female	61·7	55·6	52·4	49·9		40·6	53·0	60·3	65·6	
Education (%)					<0·001					<0·001
Junior high school	3·9	3·2	3·1	3·2		3·7	3·1	3·2	3·3	
High school	40·5	42·3	45·8	50·0		45·6	44·1	43·9	45·0	
Diploma/certificate	25·8	27·8	27·5	27·3		27·3	27·7	27·2	26·3	
University degree	29·4	26·5	23·3	19·2		23·1	24·8	25·5	25·2	
Unknown	0·4	0·2	0·3	0·3		0·3	0·3	0·3	0·3	
Income (%)					<0·001					<0·001
Low	33·0	38·5	42·7	48·4		38·5	41·0	41·3	41·8	
Medium	48·3	48·2	46·8	43·3		48·5	47·0	46·4	44·9	
High	16·1	11·0	8·4	6·0		10·7	9·9	10·0	10·8	
Unknown	2·5	2·3	2·1	2·3		2·3	2·1	2·3	2·5	
Smoking (%)					<0·001					<0·001
Never smoked	75·2	72·2	69·3	65·7		59·2	70·0	75·3	77·9	
Current smoker	6·4	9·0	11·0	12·8		16·0	9·9	7·4	5·9	
Ex-smoker	14·4	14·8	15·3	16·9		20·3	16·2	13·2	11·8	
Ever smoked but status unclear	1·4	1·7	2·0	2·2		2·3	1·7	1·8	1·4	
Unknown	2·6	2·3	2·3	2·4		2·2	2·1	2·3	3·0	
Alcohol consumption (%)					<0·001					<0·001
Occasional social drinker	53·6	58·9	62·1	62·4		61·4	60·7	59·2	55·7	
Never	32·3	26·8	23·7	21·3		19·6	25·2	27·8	31·5	
Regular drinker	3·0	4·3	5·3	6·9		9·2	4·8	3·1	2·3	
Stopped	10·0	8·8	8·1	8·3		8·8	8·3	8·8	9·2	
Unknown	1·2	1·2	0·9	12·0		1·0	1·0	1·1	1·4	
Strenuous activity (%)					<0·001					<0·001
None	45·2	46·2	45·7	43·5		50·5	46·8	44·7	38·4	
1–3 times/week	34·4	34·8	34·3	34·8		31·9	34·4	35·4	36·7	
≥4 times/week	17·8	16·3	17·6	19·1		15·2	16·3	17·4	22·0	
Unknown	2·7	2·6	2·4	2·6		2·4	2·5	2·5	2·9	
Moderate activity (%)					<0·001					<0·001
None	47·0	46·8	45·6	42·1		51·5	46·9	44·4	38·7	
1–3 times/week	33·0	34·1	34·4	35·2		31·6	34·2	35·1	35·7	
≥4 times/week	15·9	15·5	16·6	19·1		13·5	15·3	16·8	21·5	
Unknown	4·1	3·7	3·4	3·6		3·4	3·6	3·7	4·1	
Mild activity (%)					<0·001					<0·001
None	59·7	62·0	61·7	59·1		68·1	62·7	59·4	52·3	
1–3 times/week	23·1	22·4	23·1	23·3		19·2	22·8	24·1	25·9	
≥4 times/week	12·5	11·3	11·3	13·3		8·7	10·3	12·3	17·1	
Unknown	4·7	4·3	3·9	4·2		4·0	4·2	4·2	4·6	
BMI (kg/m^2^)										
Mean	21·9	21·8	21·8	21·8	<0·001	22·4	21·8	21·5	21·4	<0·001
sd	3·3	3·4	3·5	3·5		3·5	3·4	3·4	3·3	
High blood pressure (%)	5·1	4·4	4·3	4·3	<0·001	5·3	4·5	4·2	4·0	<0·001

*P values were based on ANOVA or *χ* ^2^ test.

### Dietary patterns and hypertension

The associations between the Modern and Prudent dietary pattern scores and hypertension incidence are shown in Table 2. Increasing scores on the Modern dietary pattern were associated with increased odds of hypertension in 2013 in the multivariable model (OR=1·51; 95 % CI 1·31, 1·75 for Q4 *v.* Q1, *P* for trend<0·001, respectively). Addition of BMI to the model substantially attenuated the OR estimates for the associations between the Modern dietary pattern score and hypertension (25 %).

**Table 2:** Association between dietary patterns and 8-year incidence of hypertension among adults, Thai Cohort Study (*n* 36293)

	Q1	Q2		Q3		Q4		
	OR	OR	95 % CI	OR	95 % CI	OR	95 % CI	*P* for trend
Modern dietary pattern								
Model 1	1·00	1·20	1·05, 1·37	1·39	1·21, 1·60	1·58	1·37, 1·82	<0·001
Model 2	1·00	1·18	1·03, 1·34	1·34	1·17, 1·54	1·51	1·31, 1·75	<0·001
Model 3	1·00	1·14	1·00, 1·31	1·26	1·09, 1·45	1·39	1·20, 1·61	<0·001
Prudent dietary pattern								
Model 1	1·00	0·80	0·70, 0·91	0·70	0·61, 0·80	0·84	0·73, 0·96	0·035
Model 2	1·00	0·85	0·75, 0·97	0·76	0·66, 0·88	0·93	0·81, 1·07	0·670
Model 3	1·00	0·88	0·77, 1·01	0·80	0·69, 0·92	0·99	0·86, 1·15	0·613

Model 1 adjusted for age and sex. Model 2 further adjusted for education, income, smoking, alcohol drinking and physical activity. Model 3 further adjusted for BMI in 2009 (continuous).

Having a higher Prudent dietary pattern score was associated with lower odds of hypertension incidence in 2013 in the age- and sex-adjusted model (OR=0·84; 95 % CI 0·74, 0·96 for Q4 *v.* Q1; *P* for trend=0·034). However, after further adjustment for other covariates the inverse association was not statistically significant for the high intake group.

In the full model, after excluding all those with missing values, there were 32777 participants (90·3 % of the full sample). The association between dietary patterns and hypertension did not change. OR (95 % CI) for incident hypertension were 1·00, 1·09 (0·95, 1·26), 1·22 (1·05, 1·42) and 1·35 (1·16, 1·58) across quartiles of the Modern pattern; and 1·00, 0·91 (0·79, 1·05), 0·83 (0·71, 0·96) and 1·04 (0·90, 1·21) across quartiles of the Prudent pattern.

## Discussion

In this large prospective cohort study of adults in Thailand, data collected on twelve transitional food groups plus fruits and vegetables revealed two dietary patterns: Modern and Prudent. Increasing intake of the Modern dietary pattern was positively and progressively associated with the risk of hypertension. No significant association between the Prudent dietary pattern and hypertension was found. To the best of our knowledge, this is the first longitudinal evidence on the association between dietary patterns and hypertension among adults in Thailand.

### Modern dietary pattern and hypertension

A positive association between the Modern dietary pattern and hypertension in our study is consistent with current knowledge gained in Western countries. The Modern pattern had high loadings of processed foods including roasted/smoked foods, instant foods, fermented fruits/vegetables, fermented foods and soft drinks. In Western countries there is a positive association between ‘Western’ dietary patterns and hypertension^(^ ^19^ ^–^ ^23^ ^)^, although a null association has also been reported^(^ ^24^ ^–^ ^28^ ^)^. However, in Asia no significant association between a Western dietary pattern and hypertension was found in most existing studies^(^ ^29^ ^–^ ^36^ ^)^, with a small number of studies showing a positive association between this pattern and hypertension^(^ ^37^ ^–^ ^40^ ^)^. The Western dietary pattern contains processed foods, is low in fibre and has high amounts of refined carbohydrates, fat (especially saturated fat) and salt.

The attenuation of the association between the Modern dietary pattern and hypertension that was observed after the addition of BMI to the model suggests that body size has a major impact on the association between intake of the Modern dietary pattern and hypertension. Moreover, we found after adjusting for age and sex that a high intake of the Modern dietary pattern was incrementally associated with increased BMI in a dose–response manner. It is well known that a high consumption of high-energy-density foods leads to increased BMI by increasing energy intake. In the TCS data, it has been noted that obesity is a strong risk factor for hypertension^(^ ^16^ ^)^.

### Prudent pattern and hypertension

A null association between the Prudent dietary pattern and incident hypertension in the age- and sex-adjusted model in our study differs from studies in Western countries. In Western populations, dietary patterns rich in vegetables are associated with reduced risk of hypertension or lower levels of blood pressure^(^ ^19^ ^–^ ^22^ ^,^ ^26^ ^–^ ^28^ ^)^. However, in Asian populations, the association between vegetable-rich dietary patterns and hypertension or blood pressure remains uncertain. While some studies in Asia found no association between a vegetable-rich dietary pattern and blood pressure^(^ ^32^ ^)^, other studies found inverse^(^ ^33^ ^,^ ^39^ ^)^ or positive^(^ ^30^ ^)^ associations. A ‘Traditional’ pattern with high loading of cereals, green-yellow vegetables, salted vegetables and light-coloured vegetables was not related to the risk of hypertension among Korean women aged 30–79 years^(^ ^32^ ^)^. It has been hypothesized that the cooking method for vegetables may partly explain the null association. In many Asian countries including Thailand, vegetables are often stir-fried or cooked with salt. Among Chinese adults, high intake of a vegetable-rich food pattern was positively associated with increased body size^(^ ^41^ ^,^ ^42^ ^)^. No association between a vegetable-rich food pattern and mortality was found in the Shanghai Women’s Health Study^(^ ^43^ ^)^.

### Limitations and strengths

The main limitation of the present study is the use of a short version of an FFQ without portion size. We were not able to adjust for overall energy and salt intakes. The food items selected in the study represent the ones most commonly consumed in the study population, covering both healthy and unhealthy food components. The dietary data were self-reported, which is a common method to obtain dietary intake information in large epidemiological studies. The two derived dietary patterns show the combination of food items. The second limitation is the use of self-reported doctor-diagnosed hypertension. Under-reporting is of concern as the prevalence of hypertension was lower than in studies using measured blood pressure. However, a validation study conducted with a sample of TCS participants indicated high accuracy of hypertension self-report^(^ ^18^ ^)^. We did not conduct residence-specific analyses as the multivariable models have adjusted for education and income levels. The strength of the study is the large sample size and the overall characteristics of the study population living all over Thailand with modest income and typical behaviour. As well, the prospective longitudinal study design and gathering of data on a large array of covariates allowed us to minimize the reverse causation between dietary patterns and incident hypertension across an 8-year period, a substantial portion of adult life. The study has public health significance. It gives a clear message to policy makers and health workers in Thailand that prevention of hypertension should focus on reducing consumption of the modern diet together with other measures. This strategy is now worth testing in the Thai population.

## Conclusions

The Modern dietary pattern was positively associated with the risk of hypertension among Thai adults. No significant association between the Prudent dietary pattern and hypertension was found. Promoting healthy eating by reducing the consumption of modern fast foods is important to prevent hypertension in Thailand.

## Supplementary material

For supplementary material accompanying this paper visit https://doi.org/10.1017/S1368980018002203. click here to view supplementary material
