# Supplementary material for: Dietary patterns associated with hypertension risk among adults in Thailand: 8-year findings from the Thai Cohort Study
Source: Public Health Nutr. 2018 Sep 6;22(2):307–13. doi: 10.1017/S1368980018002203 (PMC6390399; doi:10.1017/S1368980018002203)
Supplement: Supplementary file 1 [file S1368980018002203sup001.docx]

**Supplement Figure 1** Association between quartiles of dietary patterns and BMI in 2005 and 2009


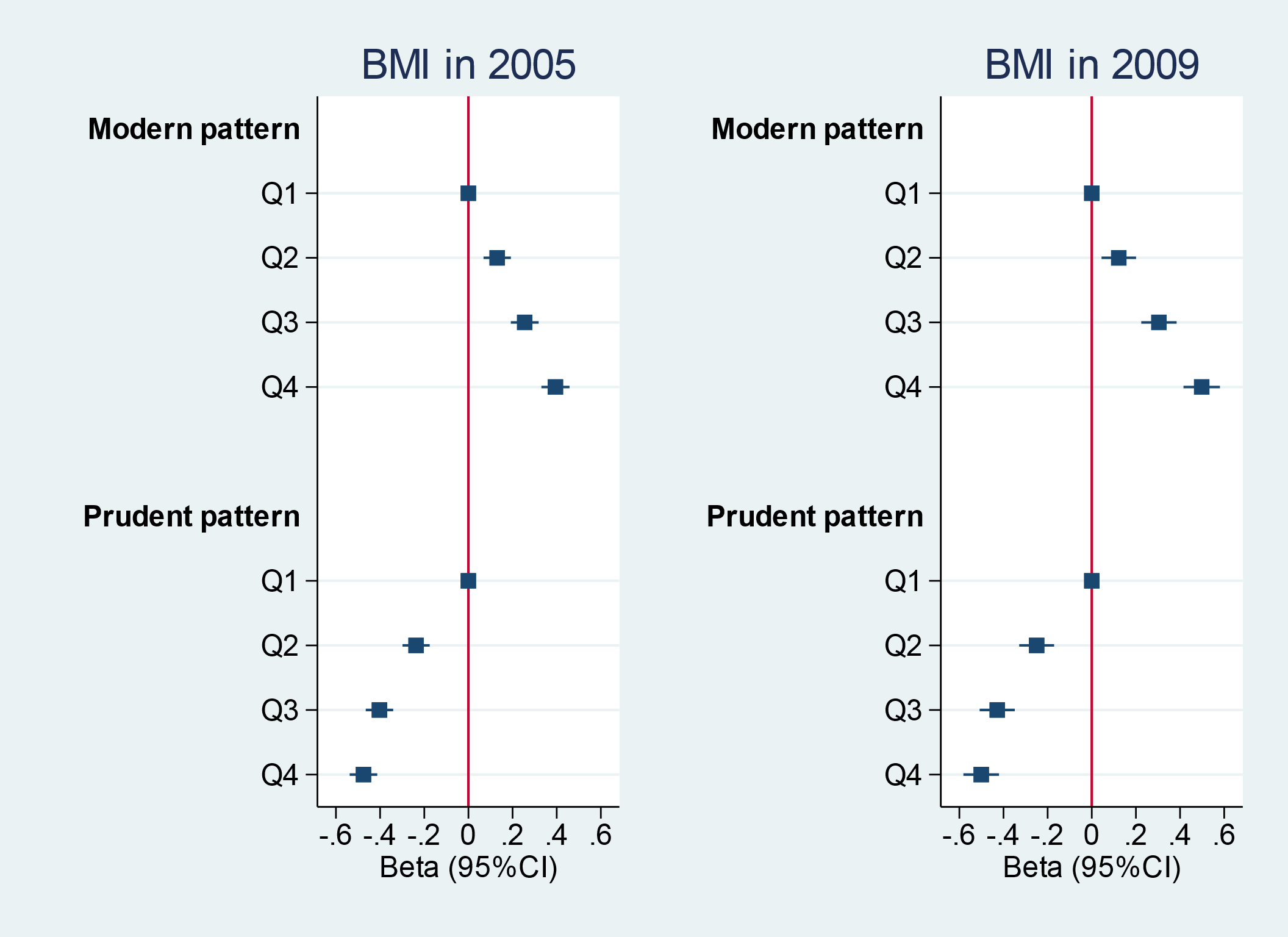


Values represent age and sex adjusted regression coefficients (Beta) and 95%CI.
